# Preservation of residual bone dimension for immediate implantation using horizontal socket shield: A case report

**DOI:** 10.6026/973206300210452

**Published:** 2025-03-31

**Authors:** Mohamad El Moheb, Mohammed Ghazi Sghaireen, Rakhi Issrani, Muhammad Nadeem Baig

**Affiliations:** 1 Private Practitioner, International Group for Oral Rehabilitaton, Beaumont sur Oise, France, Europe; 2 Department of Prosthetic Dental Sciences, College of Dentistry, Jouf University, Sakaka, Kingdom of Saudi Arabia; 3 Department of Preventive Dentistry, College of Dentistry, Jouf University, Sakaka, Kingdom of Saudi Arabia; 4 Department of Research Analytics, Saveetha Dental College and Hospitals, Saveetha Institute of Medical and Technical Sciences, Saveetha University, Chennai, India

**Keywords:** Implants, extraction, bone loss

## Abstract

Vertical bone loss post-extraction is influenced by inflammatory responses and bone remodeling. Horizontal partial extraction Therapy helps preserve bone by preventing additional resorption. A 50-year-old patient with a fractured incisor was treated using horizontal partial extraction therapy with a root form R3510 implant. One-year follow-up showed no vertical bone loss and minor implant deviation, confirming its effectiveness. This case highlights Horizontal partial extraction's potential in maintaining bone structure and calls for further research.

## Background:

Vertical bone loss following tooth extraction is a well-established phenomenon that can impact the outcomes of subsequent dental implants and restorative procedures. Studies emphasize the importance of both short- and long-term strategies to preserve bone mass and mitigate loss. In 1976, Scult and Heimke first described the technique of instantaneous implant insertion, which involves placing an implant during the same surgical session as a tooth extraction [1]. This approach offers several advantages, viz.; it reduces the number of surgical procedures, shortens the overall duration of therapy and provides the surgeon with a clear guide for implant placement, making it more convenient for the patient. However, the pattern of bone resorption that typically follows tooth extraction presents challenges and makes this technique more complex and less predictable [2, 3]. Soft tissue resorption often accompanies this bone loss, which can negatively affect cosmetic outcomes, particularly in patients with a thin biotype [4]. Preservation of bone volume following tooth extraction is crucial for the success of immediate implant placements [5, 6]. The Horizontal partial extraction Therapy has emerged as a promising method for preserving the residual bone dimensions during immediate implant placement, particularly in the aesthetic zone. The objective of Horizontal Partial Extraction Therapy is to place an implant immediately after tooth extraction to minimize or even completely halt vertical bone resorption. This report describes a case where the horizontal partial extraction therapy was used to preserve the residual bone dimension and facilitate immediate implant placement.

## Case report:

A 50-year-old patient presented with a fracture of the right maxillary central incisor. The buccal plate typically resorbs quickly following tooth extraction due to its thin buccal cortex [7]. To address this, we decided to employ horizontal partial extraction therapy to mitigate vertical bone resorption.

## After comprehensive treatment planning, the following steps were undertaken to achieve our goal:

Piezoelectric inserts were used for a non-traumatic extraction procedure to preserve the periodontal ligaments [8]. It was essential to ensure that the insert's type did not contact the first three millimeters of the residual root. Cone-beam computed tomography (CBCT) scan was taken following the extraction to ensure the length of the residual root. Following extraction, the root was sectioned and hollowed out in its cervical portion by 3 mm. The root form implant R3510 from Trate was selected for this procedure. This implant was chosen for its tapered shape, which significantly enhances stability [9]. The implant includes an abutment designed to accommodate the root ring. The implant was placed without raising a flap, positioned 3 mm below the cementoenamel junction of the adjacent tooth [10]. A horizontal shield, made from the prepared root segment, was attached to the implant. To protect the screw head, a Teflon piece was placed inside the abutment and sealed with flowable composite. No bone graft or collagen was used; stabilization of the clot inside the socket was achieved through natural healing processes. One year later, at the recall, a cone-beam computed tomography was made to check the buccal bone level; we found that there was no vertical bone loss. The implant was little bit deviated but still there was no bone resorption.

## Discussion:

In the field of restorative dentistry and dental implants, vertical bone loss following tooth extraction is a frequent concern. For the implants to be successful and for general oral health, it is imperative to comprehend and treat this phenomenon with utmost care for more predictable and sustainable treatment. There are well-documented reasons and causes of vertical bone loss such as [11].

[1] Immediate post-extraction changes such as alveolar ridge resorption which is frequently caused by the loss of the root structure, which otherwise helps to keep the volume of the bone intact. Also numbers of inflammatory response which occur during the healing phase exacerbate vertical bone loss.

[2] Long-term bone remodeling may result from missing teeth. The degree of infection, the state of the bone and an individual's healing reaction are some of the factors that may affect this process.

Thus, the inflammatory response that underlies bone resorption is a complicated series of biochemical cascades that include matrix metalloproteinases, cytokines and markers of bone remodeling such blood osteocalcin and urine deoxypyridinoline. Developing efficient therapeutic strategies to reduce vertical bone loss requires an understanding of these mechanisms at the histology and biochemical levels [12]. The stability and success of dental implants can be strongly impacted by vertical bone loss following tooth extraction. Comprehending this correlation is essential for proficient implant design and implementation. In implant dentistry, a horizontal socket shield can be used specifically in implant therapy that involves socket preservation. It is intended to preserve the bone's structure and stop resorption in the socket area following tooth extraction, facilitating a successful implantation. Resorption of the socket following tooth extraction may result in a decrease in the volume of bone accessible for upcoming implants. Maintaining this volume is aided by a horizontal socket shield. It also guarantees that there will be sufficient bone to firmly place the dental implant in the future by maintaining the density and contour of the bone and it helps protect the socket from the forces that could cause further resorption. According to previous research that has been referenced with background knowledge, closing the wound as quickly as feasible is the goal of the healing mechanism. To get the soft tissue closed at the extraction site for healing, the body initiates a vertical bone resorption. Now, there is no need for the body mechanism to produce vertical bone resorption to close the wound if the surgical site signals that it is already closed. Repositioning the root in its socket facilitates a seal by fibrous reattachment, as shown by the research of Huston *et al.* [13]. After the root is extracted, putting in place a horizontal shield that permits fibrous attachment could signal to the healing process that the wound has closed and halt bone resorption. Innovative methods, such as horizontal socket shields, have been developed to preserve the horizontal dimensions of the alveolar ridge following tooth extraction. Over time, various materials have been investigated for this purpose like:

[1] Natural tooth socket shield: has long been one of the first choices as this approach has the advantage of seamlessly integrating with the surrounding bone by keeping a piece of the natural tooth's root or crown inside the extraction socket. However, careful planning and execution are needed to ensure perfect integration and prevent difficulties [14].

[2] Bioactive glass socket shield: product blends in fluidly with natural bone and can encourage bone regeneration. Although bioactive glass contains osteoconductive qualities that promote bone repair, it must be managed carefully to guarantee correct integration and prevent tissue overgrowth [15].

[3] Collagen membrane socket shield: is positioned in the socket horizontally to maintain the shape of the ridge. Because collagen membranes are biocompatible and promote soft tissue healing, they are frequently utilized in combination with other graft materials. It can be used in conjunction with bone grafts to improve preservation overall. However, to guarantee sufficient ridge retention, the shield could need extra support from bone grafts [16].

[4] Resorbable synthetic socket shield techniques with polyglycolic acid or polylactic acid: as two examples have been widely used for their resorbable properties. Over time, natural bone gradually replaces these components as they degrade. When new bone gradually replaces the old, these components offer interim support to maintain the structure of the bones. They save the necessity of taking off the shield. To guarantee optimal bone preservation, special attention must be paid to the requirement that the rate of resorption match the healing process [17].

[5] Customized titanium mesh: shield is designed to fit the extraction socket, serving as a physical barrier to maintain the ridge's dimensions while offering robust support. To avoid issues like exposure or infection, the titanium mesh must be carefully placed and monitored. It can be adjusted to fit à variety of socket shapes and sizes [18].

These various types of horizontal socket shields provide different advantages depending on the clinical situation and specific needs. Often used in combination with bone grafting, guided bone regeneration, or immediate implants to maximize outcomes coupled with regular monitoring and follow-up is essential to ensure the shield's effectiveness and to address any complications.

Biocompatible materials that integrate are usually used to make the shield. In this case, the authors are promoting the usage of the root ring or the root shield. Authors have tried to address this issue by using natural tooth as horizontal socket shields as they are most biocompatible, they allow connective tissue re-attachment and they preserve a natural emergence profile, concurrently carrying advantage of minimal material cost for the patients. It is imperative that this root ring must be at least 3 mm thick to fully cover the cervical space for the implant platform. Based on the study by Chen and Buser, 3 mm thickness is chosen as the implant comfort zone is 3 mm under the cementum junction of the adjacent tooth, which favors a positive outcome [4].

The implant stability is very important because this horizontal partial extraction therapy is a sort of immediate loading. Once this shield is placed and stable it will secure a sealing by the fibrous tissue and it will secure a natural emergence profile for the future prosthetics. It has been demonstrated that horizontal socket shields are useful in retaining the horizontal proportions of the alveolar ridge, which can be important for keeping implant stability and producing aesthetically pleasing outcomes. Present case study reinforces the knowledge that using horizontal socket shields like horizontal partial extraction therapy in conjunction with prompt implantation can result in positive long-term effects, such as decreased bone resorption and stable implant integration. Various methods have been described by various authors and applying socket shields may have an impact on how well the ridge is preserved. Shalimon *et al.* found that delayed implant loading resulted in greater osseodensity around the buccal aspect of the implant compared to the immediate loading protocol, as evaluated using cone-beam computed tomography [19]. Authors strongly advocate studying comparative studies to determine which approaches work best in different therapeutic contexts.

## Conclusion:

Horizontal partial extraction therapy with timely implant placement reduces implant instability and bone resorption. The therapy helps minimize vertical bone loss after extraction, but further research is needed. Future studies focusing on comparative research for wider clinical use are needed.
